# Transcriptomic analysis of the exit from dormancy of *Aspergillus fumigatus *conidia

**DOI:** 10.1186/1471-2164-9-417

**Published:** 2008-09-16

**Authors:** Claude Lamarre, Sergueï Sokol, Jean-Paul Debeaupuis, Christine Henry, Céline Lacroix, Philippe Glaser, Jean-Yves Coppée, Jean-Marie François, Jean-Paul Latgé

**Affiliations:** 1 Unité des Aspergillus, Institut Pasteur, 25 rue du Docteur Roux, 75724 Paris Cedex 15, France; 2 Plateforme Biopuces, UMR 5504 & 792 d'Ingenierie des Systèmes Biologiques et Procédés, INSA-CNRS-INRA, 135 Avenue de Rangueil, 31077 Toulouse Cedex 04, France; 3 Plate-forme Puces à ADN, Institut Pasteur, 28 rue du Docteur Roux, 75724 Paris Cedex 15, France; 4 Laboratoire de Génomique des Microorganismes Pathogènes, Institut Pasteur, 28 rue du Docteur Roux, 75724 Paris Cedex 15, France; 5 Unité de Biologie et Génétique du Paludisme, Institut Pasteur, 28 rue du Docteur Roux, 75724 Paris Cedex 15, France

## Abstract

**Background:**

Establishment of aspergillosis is depending upon the exit from dormancy and germination of the conidia of *Aspergillus fumigatus *in the lung. To gain an understanding of the molecular mechanisms underlying the early steps of conidial germination, we undertook a transcriptomic analysis using macroarrays constructed with PCR fragments from > 3,000 genes (around one third of the annotated *A*. *fumigatus *genome).

**Results:**

Major results of this analysis are the following: (i) conidia stored pre-packaged mRNAs transcripts (27% of genes have transcripts in the resting conidia; (ii) incubation at 37°C in a nutritive medium induced up- and down-regulation of genes: 19% of the total number of genes deposited on the array were up-regulated whereas 22% of the genes with pre-packaged mRNA in the resting conidia were down-regulated; (iii) most modifications were seen during the first 30 min of germination whereas very little modification of gene expression occurred during the following hour; (iv) one-year old conidia and one-week old conidia behaved similarly at transcriptional level.

**Conclusion:**

Transcriptomic data indicate that the exit from dormancy is associated with a shift from a fermentative metabolism to a respiratory metabolism as well as a trend toward immediate protein synthesis.

## Background

Conidia represent the main reproductive route of the opportunistic fungal pathogen *Aspergillus fumigatus *[1]. Metabolically inactive, the conidium will produce a new individual by responding to an environmental stimulus, through reactivating the cell biochemical and genetic machineries, a process called germination. Germination of the conidia of *A. fumigatus *in the lung is a key step in the establishment of aspergillosis. Conidial germination can be divided into four stages: (i) breaking of spore dormancy; (ii) isotropic swelling; (iii) establishment of cell polarity and (iv) formation of a germ tube and maintenance of polar growth [2,3]. Although conidial germination is a crucial step in the life cycle of all filamentous fungi, it is surprising that the basic molecular steps are poorly understood [1]. Studies focused on the analysis of this process have been very limited, and most of them referred to stages ii to iv.

With the exception of some species like *Neurospora crassa*, in which germination occurs sporadically in the absence of a specific growth stimulus, germination is initiated when conidia are supplied with the appropriate nutrients (usually fermentable sugars or other carbon sources as acetate, glycerol and starch) in the presence of water and air. This is the case for the *Aspergillus *species analysed to date [1]. The nutrient signal is recognized by one or many unknown receptors, and transmitted throughout several signal-transduction pathways. In spite of conflicting data, the ras/MAPK and cAMP/PKA pathways seem the main signalling pathways involved in the conidial germination of *Aspergillus *[4-9].

In *Aspergillus nidulans*, activation of these pathways leads to the breakdown of the trehalose and the synthesis of glycerol or/and mannitol, concomitant with an increase of the turgor pressure responsible for the swelling of the conidia [10]. Protein synthesis is associated to conidial germination whereas RNA synthesis is apparently not necessary [6]. Those results have suggested the presence in the dormant conidium of a pre-existing pool of mRNAs that are rapidly translated in the presence of nutrient. Such stored mRNAs were shown to be present in *A. nidulans *conidia by Timberlake [11] and Zimmermann and co-workers [12], but none were identified by these authors. Osherov and co-workers [13] have identified twelve genes with transcripts that are stored in *A. nidulans *resting conidia. Due to the sequencing of genomes of filamentous fungi, it is now possible to undertake a comprehensive analysis of the conidial mRNAs since they may reveal which proteins need to be translated at the very beginning of germination and control conidium dormancy, an essential event for a general understanding of the biology of filamentous fungi and more generally of key events in the eukaryotic life.

This work is aimed at understanding molecular mechanisms underlying conidial exit from dormancy in the opportunistic pathogen *A. fumigatus*. In addition to the fundamental interest mentioned above, advances in the field should also have applied benefits in fungal infectiology since for example in *A. fumigatus*, exit from conidial dormancy and subsequent germination are essential for the establishment of this opportunistic pathogen in patients lung. By hybridizing DNA macroarrays with [α-^33^P]dCTP-labelled cDNA obtained from young and/or old conidia, after incubation in a nutritive medium at 37°C for up to 90 min, we could identify genes differentially expressed in the very first molecular events occurring during the germination process.

## Results

### Conidial germination and dormancy

**Figure 1:**
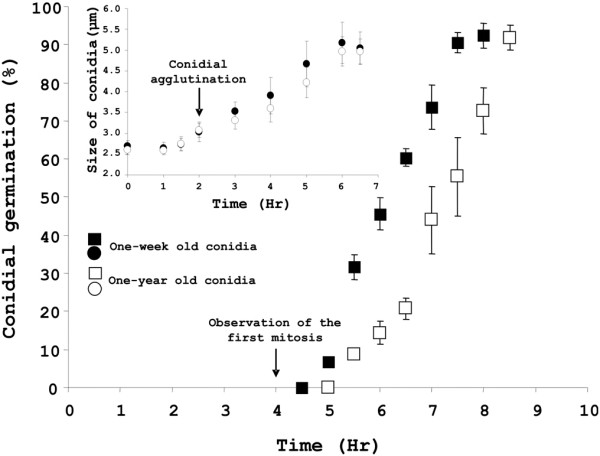
**Germination kinetics of one-week and one-year old conidia.** Swelling of the conidia and germ tube formation is computed. Agglutination of swollen conidia and first doubling of the nucleus is shown by an arrow.

A kinetic study of germination of conidia incubated at 37°C in YPD is shown in figure 1. No morphological modification of the conidium was observed during the first 90 min of incubation in the culture medium. After 2 h of incubation, conidia started agglutinating. This event was concomitant with conidial swelling. These morphological changes were associated with loss of refringency of the conidia. Conidia continued to swell overtime and the first germ tube (polarized growth) was seen after 4 h of incubation in the culture medium. This was concomitant with the first mitosis. Eight hours after the beginning of incubation, all conidia had produced a germ tube. Incubation into a nutritive medium was required for germ tube formation because conidia suspended in water failed to germinate but remain viable without any morphological changes (results not shown).

Resting conidia were able to survive after at least one year of storage at room temperature. No changes in the early stages of germination were seen and only a slight delay in the germ tube formation was seen between one-week old and one-year old conidia batches (Fig. 1).

### Identification of stored mRNA in dormant conidia

In order to quantify and identify genes having stored mRNA in dormant conidia, macroarray membranes were probed with [α-^33^P]dCTP-labelled cDNA obtained from one-week and one-year old conidia. Membranes hybridization showed that 27% of genes (844/3,182) had a detectable transcript (see Additional file1^1^ ). Among these 844 genes, 279 (33%) were annotated as hypothetical proteins.

**Figure 2:**
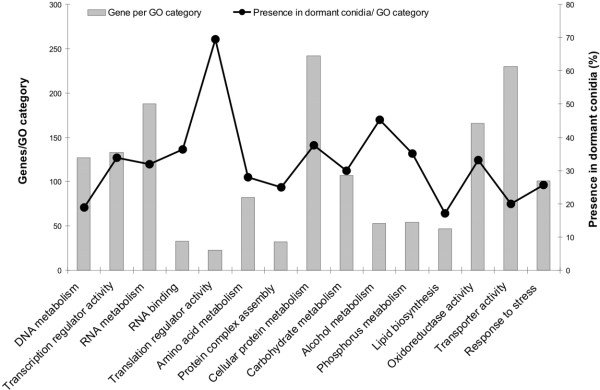
**Putative molecular function or biological process (matched to the Gene Ontology) of genes with an active transcripts in dormant one-week and one-year old conidia.** Histogram indicated the total number of genes of the GO category deposited on the membrane, and the curve the percentage of genes per GO category with transcript stored in the resting conidia.

The GO functional category repartition is illustrated in figure 2. All functional categories of genes were found in the pool of transcripts present in resting conidia. Some categories however were present in higher proportion in the conidia: translation regulatory activity, RNA binding, alcohol and phosphorus metabolisms. There was not a higher amount of developmental and sporulation genes in the conidium that would be indicative of deposition of mRNAs during the process of asexual sporulation. In contrast, transport activity, lipid biosynthesis and DNA metabolism were rather under represented in the dormant conidia. A variation of 10% in the total number of genes with mRNAs at time 0 was seen between young and old conidia (data not shown). This result indicated that the same mRNAs were present in similar concentration in one-week and one-year old conidia.

### Prominent transcriptional changes occurring during the first 30 min of incubation of conidia in a rich medium

**Figure 3:**
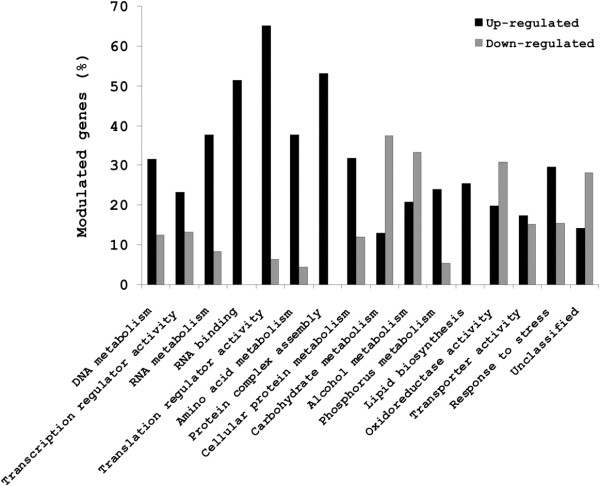
**Putative molecular function or biological process (as GO terms) of genes up- and down-regulated during the first 30 min germination of young conidia.** Modulated expression of up-regulated genes = number of up-regulated genes/total number of genes deposited on the array × 100; modulated expression of down-regulated genes = number of down-regulated genes/total number of genes with an active transcript at T0 × 100.

In the first 30 min of incubation of one-week old conidia in YPD at 37°C, a total of 787 genes (25%) were differentially expressed, in which up- and down-regulated genes accounted respectively for 598 (19%) and 189 (6%) genes (see Additional file 2). The 598 up-regulated genes originated from 3,182 genes deposited on the array and represented 19% of these genes whereas the 189 down-regulated genes came from the 844 genes that had a pre-packaged mRNA in the dormant conidium and accounted for 22% of these genes. The functional categories of these genes are shown in figure 3 (see also Additional file 3), and genes are listed in Additional file 4. Down-regulated genes belong mainly to fermentative metabolism and oxidoreductase activity whereas up-regulated genes were over-represented within the RNA and phosphorus metabolism, amino acid and protein biosynthesis and protein complex assembly.

**Figure 4:**
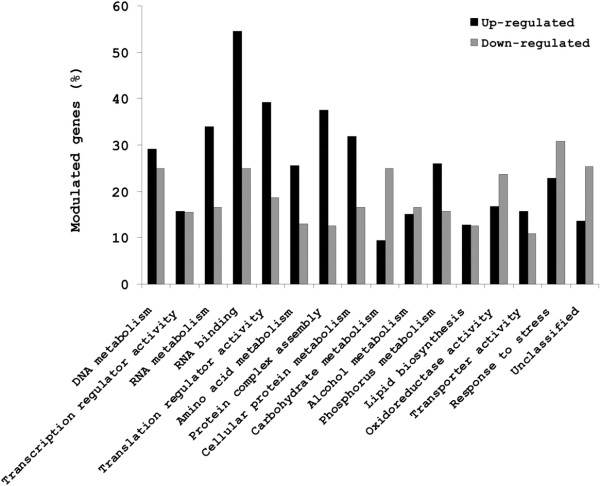
**Putative molecular function or biological process (as GO terms) of genes up- and down-regulated during the first 30 min germination of one year-old conidia.** Same legend as in Fig.3.

The regulation of gene expression of one-year old conidia upon germination was similar to the one of one-week old conidia (Fig. 4 and Additional file 3). A total of 734 genes (22%) were differentially expressed, in which up- and down-regulated genes accounted respectively for 542 and 192 genes. The same GO families were up- and down-regulated in young and old conidia, in agreement with the same capacity of the young and old conidia to germinate (see Additional files 5 and 6 for a detailed list of the up- and down-regulated genes during germination of the old conidia and their repartition among the different GO families).

Many of the genes that were up-regulated had already stored transcripts at time 0 (38 and 46% for young and old conidia respectively). The genes *de novo *up-regulated shared however the same GO families as the one of genes with a stored transcript at time 0 (data not shown).

### Specific transcriptional changes associated to early conidial germination

Analysis of the genes which transcripts varied during the first 30 min of germination was focused on the genes which expression varied the most significantly during early germination of both one-week and one-year old conidia. Such analysis allowed the recognition of specific pathways and biochemical events associated with conidial germination.

#### Protein synthesis and amino acid metabolism

Genes that function in transcription, purine and pyrimidine biosynthesis, protein synthesis and post-translational modification, ribosome biogenesis were highly activated. Among the 40 transcription factors up-regulated during germination (see Additional file 7), *ZPR1 *(Afu6g10470) was the most up-regulated. *ZPR1 *is a zinc finger protein that localizes to the nucleolus of yeast cells growing in log phase, redistributes to the cytoplasm when cells are deprived of glucose and translocates to the nucleus when glucose is restored [14,15]. Genes that are involved in cell cycle/DNA processing and initiation of DNA replication were not over-represented after 30 min of germination. This was in agreement with the cytological data of figure 1 showing that the first nuclear division occurred after 4 hours of incubation in YPD medium. In agreement with the requirement of a rapid protein synthesis (that requires a very quick use of the free pool of amino acid), expression of genes involved in amino acid synthesis (lysine, leucine, valine, phenylalanine, threonine, histidine, isoleucine, arginin, glutamic acid) was up-regulated during the first 30 min. Interestingly, the two genes of the aminoadipic pathway, the homoaconitase (Afu5g08890) and homocitrate synthase (Afu4g10460), involved in the synthesis of lysine in fungi, showed an increased expression suggesting a specific requirement of lysine in *A. fumigatus *proteins during germination. Genes coding for enzymes responsible for isopropylmalate (Afu1g15000) and chorismate synthesis (Afu1g06940) that are intermediates of the biosynthesis of leucine and aromatic amino acids were also highly expressed. In agreement with this activation of the amino acid synthesis pathway, no gene coding for amino acid permeases was over-expressed. Among the seven amino acid permease genes of *A. fumigatus *deposited on the array (17 genes are reported in the genome), only one (Afu3g01560) was up-regulated during germination.

Increasing expression of the genes coding for glutamate dehydrogenase (Afu2g06000) and glutamate synthase (Afu1g07380) suggest an active coupling of the carbon and nitrogen flux through the 2-ketoglutarate towards amino acid biosynthesis. The NADPH isocitrate dehydrogenase (Afu1g12800) that produces the 2-ketoglutarate was also highly activated. Succinate dehydrogenase (Afu3g07810), also coupling TCA cycle to amino acid synthesis through the intermediary of 2-ketoglutarate was up-regulated.

#### Cellular transport

Although the analysis of the modulated expression of genes did not indicate a significant increase in the expression of transporters (Fig. 3), a survey of the most highly expressed genes indicated that many cellular transport systems are induced in germinating conidia (see Additional file 3). Among the 40 up-regulated genes on a total of 230 deposited on the array, were a putative MFS transporter (Afu1g03200) and the plasma membrane H^+^-ATPase *PMA1 *(Afu3g07640). *PMA1 *is an essential gene that encodes the major plasma membrane H^+^-ATPase in *Saccharomyces cerevisiae *as in *A. fumigatus *[16]. By hydrolyzing ATP, Pma1p pumps H^+ ^ions out of the cell and drives the secondary import of nutrients across the plasma membrane. Pma1p is highly regulated by glucose, both transcriptionally and post-translationally. The presence of glucose induces a 2- to 4-fold increase in *PMA1 *expression in *S. cerevisiae*. In *A. fumigatus*, 3 genes (Afu1g02480, Afu3g07640 and Afu5g03550) encode orthologs of *S. cerevisiae PMA1*. The three PMA1 orthologs were present in the arrays but only the expression of Afu3g0764 varied over time, suggesting that like in yeast, one of the Pma1 proteins (Afu3g07640) plays an important role whereas the others do not. In addition, induction of the genes encoding ten mitochondrial translocases and carrier proteins importing protein from the cytosol to mitochondria, was seen during germination whereas only a unique putative carrier protein was repressed (Afu2g13870).

#### Energy

Genes coding for alcohol dehydrogenase (Afu5g06240, Afu5g03930, Afu5g00300, Afu7g04530), lactate dehydrogenase (Afu4g07050), pyruvate decarboxylase (Afu6g00750) and phosphoenol pyruvate synthase (Afu5g14790) were down-regulated. In contrast, genes that function in TCA cycle were up-regulated. Similarly, 28 mitochondrial genes were up-regulated whereas only one was down-regulated. These results suggested a shift from a fermentation metabolism when the conidium is in a dormant stage to a respiration metabolism as soon as the germination process has started. The anaerobic degradation of glucose is a very low energy process. Many oxygenases are also down-regulated during this early stage, in agreement with an increased requirement for electron acceptors. Interestingly, the down-regulation of succinyl-CoA synthetase (Afu4g00290) suggested however the absence of ATP formation during succinylhydrolysis at the TCA cycle level in favour of succinate production. Although the shift towards a respiratory metabolism during germination was not associated to a global up-regulation of reductase genes, the thioredoxin reductase (Afu4g12990) and the arsenate reductase (Afu5g15000, ortholog of ArsC), that are members of a thio-disulfide exchange complex responsible for the production of reducing equivalent in bacteria [17], were up-regulated with a 5- to 20-fold ratio. This result suggested that these two enzymes may play a major role in protecting the germinating conidia from damages putatively resulting from reactive oxidant intermediates occurring during germination.

#### Cell wall modification

Swelling of the conidium is associated both with water entrance and plasticization of the conidium cell wall. The cell wall skeleton of *A. fumigatus *is mainly composed of α- and β-glucans, chitin and chitosan. Accordingly, there is a down-regulation of genes coding for glycosylhydrolase activities able to degrade cell wall polysaccharides: chitosanase (Afu6g00500), α1,3-glucanases (Afu7g08350, Afu8g06030), class III chitinase and N-acetylglucosaminidase (Afu5g03850, Afu8g04060), endoglucanase (Afu2g09520). Interestingly, no annotated β1,3-glucanase had an expression that varied overtime. None of the genes coding for glycosylhydrolases able to degrade cell wall polysacccharides deposited on the arrays were up-regulated, suggesting that their transcripts were immediately translated into enzymatic proteins softening the cell wall during conidial swelling and were not required during further steps.

#### Conidiation transcripts

It was expected that some of the transcripts seen in dormant conidia were associated with the conidiation process. This may be the case for the two transcription factors *WETA *(Afu4g13230) and *ABAA *(Afu1g04830) that have been shown to be involved in conidiation [18]. However *ABAA *transcript remained present during conidial storage at a high level and was also expressed during germination, suggesting it played a role in biological pathways other than conidiation [19]. Osmoregulation and trehalose synthesis have also been recognized as hallmark of conidium formation. Trehalose biosynthesis occurs through two different pathways in filamentous fungi. In the first one, UDP-glucose is linked to glucose-6-phosphate to form trehalose-6-phosphate. The phosphate is then removed by trehalose phosphatase. In the second one, trehalose is produced directly from glucose-1-phosphate [20-23]. Among the seven genes involved in trehalose-6-phosphate trehalose biosynthesis pathway, four genes were in the array (Afu4g03190, Afu3g05650, Afu2g04020 and Afu7g03940), among which two genes had transcripts stored in the conidia (Afu3g05650 and Afu7g03940). Up-regulation, near the threshold limit, was only seen for the gene Afu3g05650. Three trehalase phosphorylase genes (Afu3g12100, Afu5g14780 and Afu6g03420) of the glucose-1-phosphate pathway exist in *A*. *fumigatus *and one (Afu6g03420) was spotted on the array. This *CCG9 *ortholog was highly expressed in young conidia and disappeared during the 30 min incubation to reach a level similar to the one seen in 1 year-old conidia, suggesting that *(i) CCG9 *of *A. fumigatus *was a typical conidiation gene, and *(ii) *the glucose-1-phosphate pathway may be the major pathway in the production of trehalose by *A. fumigatus*. Osmoregulation could also be associated with glycine betaine in the conidium since a high expression of choline oxidase (*CODA*; Afu8g04090) was found in young and old conidia and was up-regulated during germination [24,25]. However like trehalose, glycine betaine which is the reaction product of the choline oxidase, may be involved in stabilizing membrane and protecting the transcriptional and translational machineries [26].

### Most of the transcript regulation occurs during the first 30 min of germination

Between 30 and 60 min and between 60 and 90 min, we observed changes in expression for 176 and 109 genes respectively (see Additional file 8). This was significantly lower than the 787 genes up- and down-regulated between 0 and 30 min. These results indicated that molecular responses to induction of germination are very fast and that the most significant transcriptional changes occurred during the first 30 min of germination.

**Figure 5:**
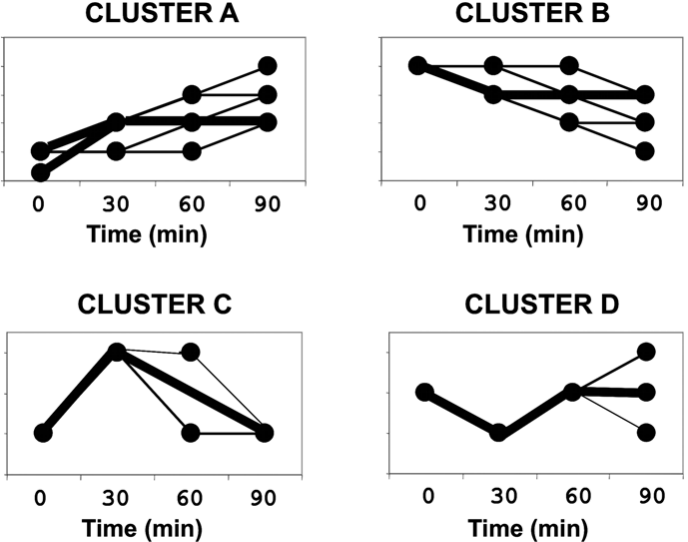
**Different patterns of gene expression seen during the first 90 min of incubation of one-week old conidia in YPD medium.** In bold is the main trend of the expression of the variation seen in the cluster.

Four expression patterns (A-D) were identified during the 90 min incubation in YPD medium (Fig. 5). Most of the genes belong to two main groups, A and B that contained most up- and down-regulated genes (see Additional file 9).

Genes that partitioned into cluster A (594) were over-expressed during the 90 min studied. Almost all genes (524) of this cluster were already up-regulated during the first 30 min of induction, with a very few showing an increased level of expression over the remaining 60 min. Few genes (24) maintained a constant up-regulated expression after 60 min and/or 90 min. It was found among the latter the thaumatin-like protein (Afu8g01710) described by the Osherov's group which expression increased over the 90 min experimented [13]. Among the genes that were not up-regulated between 0 and 30 min, only 15 genes had a level of expression regulated by a factor 3 between 30 and 60 (10 genes) and 60 and 90 min (5 genes; see Additional file 8). At the 90 min, it was interesting to note that several new genes coding for proteins involved in cell wall modification were expressed (Afu3g13200, Afu1g16190, Afu5g14380, Afu6g09310). Some of these proteins as well as the protein encoded by Afu2g03830 coding for allergen Asp F4 display antigenic properties [27].

Cluster B contains genes (203) that are down-regulated over the 90 min period analysed. The majority (176) was already regulated during the first 30 min. Nineteen and three genes were down-regulated by a factor > 2.5 respectively between 30–60 and 60–90 min intervals. No specific gene families appeared in the last stage of the incubation that just confirmed a down-regulation of the fermentation pathways.

Cluster C grouped a subset of 74 genes with expression that increased transiently in the initial 30-min-time interval, and then decreased for the remaining 60 min of this study. Cluster C contained many genes somehow associated with the TOR signal transduction pathway, an eukaryotic sensor of the environment that links nutrient assimilation with translation initiation and ribosomal protein biosynthesis [28]. Among the genes putatively associated with TOR we can cite the GATA (Afu6g01970) and forkhead transcription factors (Afu3g10030) or genes of the leucine biosynthesis (Afu1g15000, Afu2g11260, Afu1g15780). The need for intracellular detoxification was also suggested by the up-regulation of MFS (Afu1g03200) and ABC drug efflux pumps (Afu6g04360) and other transporters, thioredoxin (Afu4g12990) or arsenate reductase (Afu5g15000).

Cluster D encompassed thirteen genes down-regulated during the first 30 min of study, and then over-expressed for the remaining 60 min. Cluster D is enriched in genes coding for retrotransposons elements: LINE like reverse transcriptase (3 genes; Afu4g00820, Afu4g02640 and Afu8g01040) and transposase from either Fot5 or Taf1 transposon (Afu3g13860, Afu5g09820 and Afu6g03650) and a RNA binding protein (Afu1g02510). This result suggested that transposable elements were associated with germination.

### Confirmation of macroarray results

We have confirmed that the modifications in gene expression did not only result from a shift of conidia to a different physico-chemical environment *viz. *from dry air conditions to a shaken liquid medium at 37°C. For that purpose, the expression pattern obtained with conidia incubated in water at 37°C in shaken flasks was investigated. Overall, only 44 genes were identified as differentially expressed over the 90 min incubation (in comparison with the > 700 genes up- and down-regulated during the same time in a nutritive medium). In addition, the variation in the expression of all genes identified was very close to the significant twice threshold limit accepted for a differential expression (data not shown). This result showed that incubation of conidia in water at 37°C did not result in gene activation in agreement with the absence of conidial germination in water. It confirmed *a posteriori *that the variations of gene expression seen after incubation in the YPD culture medium were representative of the molecular machinery necessary for conidial germination.

Consistency of microarray data with northern blot and RT-PCR analysis was confirmed with selected examples belonging to the different clusters of expression seen in figure 5. For Northern assays, total RNAs were purified from conidia sampled every 5 min for 90 min (see Additional file 10). The expression profiles seen with RNA gel blots showed that major changes in gene expression followed the three major patterns seen in figure 5: (1) Disappearance of conidial transcripts after 30 min; (2) Transcripts that continue to be up-regulated until the maximal time considered, viz. 90 min; (3) Up- or down-regulation of transcripts occurring only during the first 30 min. No unique changes of expression were specifically seen before 30 min (data not shown). This analysis validated *a posteriori *our window of time and showed that the selection of a 30 min time as a first step in the analysis of the variation of gene expression during early steps of germination was appropriate.

Similarly, the expression data was verified by 74 RT-PCR reactions (see Additional file 11). The RT-PCR results coincided with the transcriptome data on 68/74 reactions tested demonstrating an acceptable 10% error between the two methods.

In addition, Western blots have shown a good correspondence between the transcriptome data and the proteome data since Pma1p was detected in high amounts only in the germinated conidia (see Additional file 12).

## Discussion

Transcriptome studies in *A. fumigatus *only started recently due to the completion of the genome of this fungal species in 2005 [29]. Global gene expression analysis of this fungus under stress conditions (thermic shock or drug) or in mutants have been already reported [29-32]. This is the first transcriptomic study of the exit from dormancy of *A. fumigatus *conidia undertaken before completion of the genome sequencing. Analysis of gene expression during germination with microarrays has been performed in several filamentous fungi (*Fusarium; Trichophyton; Neurospora; Dictyostelium, Ustilago*) [4,33-36]. It is however difficult to compare all the transcriptional data obtained to date, since these studies deal with very different fungal species or with different time intervals from early incubation time to conidial swelling and further germ tube formation. Long incubation times have not been undertaken in our study because incubation time > 2 h is associated with asynchronous growth in *A. fumigatus*. In *Dictyostelium discoideum*, global gene expression has confirmed that the first hour and later 2–6 hours of germination are characterized by the expression of different gene clusters [35]. Accordingly, the only study that can be compared to ours is the analysis of *N. crassa *germination since gene expression is analysed at 0.5 and 1 h post incubation [4]. Both studies showed that conidia apparently dormant because of an absence of morphological modifications become biochemically active as soon as they are incubated into a nutritive medium. In spite of the different experimental conditions used for the conidial germination of the fungal species reported in litterature, the percentage of gene with modulated expression during this cellular process is very similar between species, and is in the range of 1/5 of the fungal genome (15, 20 and 25% of the genes in *Trichophyton rubrum, N. crassa *and *A. fumigatus *respectively). In addition, the four types of gene expression pattern described as A-D in our study, have been seen in all the transcriptomic studies of conidial germination previously carried out.

Several studies have shown that transcripts are present in resting conidia. These transcripts could have several functions: (i) they could be absolutely required for spore metabolism during dormancy; it has been shown for example that an ongoing translation and transcription are essential for long term survival of *S. cerevisiae *spores [37]; or (ii) some of the mRNAs pre-packaged in the conidium may be conidiation transcripts that can decay overtime during conidial ageing without any consequence on conidial survival or germination [35]. In *A. fumigatus *however, most transcripts present at time 0 persisted in the conidium after one year of storage at room temperature; (iii) the third category of genes includes those that are primed for rapid activation and translation in the presence of nutrients. Genes coding for glycosylhydrolases acting on the conidium cell wall can belong to such category. Ribosomes and mRNA stored in dormant conidia allow also the conidia to start protein biosynthesis immediately after incubation in the nutrient medium. Protein synthesis is indeed one of the earliest measurable biochemical changes occurring in germination [10].

The presence of a percentage of transcripts in the *A. fumigatus *conidia representing more than 25% of the total genome has been documented here for the first time. Such data also ask the question of the mechanisms holding these huge amounts of untranslated transcripts. Dormancy is an event occurring from lower eukaryotes to higher eukaryotes including plant and mammal. In most systems, the changes (if any) occurring after growth cessation to enter dormancy and maintain dormancy are unknown [38].

Transcript of genes encoding fermentation pathway have been identified in the dormant conidium. Transcriptome data must now be completed by supplementary biochemical data to characterize this dormancy metabolism. In *A. fumigatus *our data suggested that dormancy is associated with fermentation and reduced metabolism. In fish surviving anoxia for a long time the metabolism switches from aerobic metabolism to a reduced level of anerobic metabolism. Reduction in ATP demand is the only way to survive as observed with a reduction in ion pumping which is extremely ATP-consuming. Lactate produced by the metabolism of glycogen is converted to ethanol by lactate dehydrogenase, pyruvate dehydrogenase and alcohol dehydrogenase. Ethanol diffuses easily from the gills avoiding the build up of lactic acid [39]. Basically, the situation in *A. fumigatus *is very similar with a reduction in the activity of ATP pumps and an anaerobic metabolism. Similarly, in yeast, mitochondria contribute to ageing through their propensity to generate reactive oxygen species [40,41]. Mitochondria dysfunction induces the retrograde response that activates genes compensating this dysfunction [42]. A central gene of this pathway is *RTG2*. Two orthologs of this gene were annotated in the *A. fumigatus *genome: Afu6g08350 and Afu8g00730, the later one being on the membrane. This *RTG2 *ortholog is highly expressed at time 0 and repressed by a factor 4 when conidia were incubated with nutrients, a pattern similar to the one observed in yeast, suggesting that the *RTG2 *pathway could play an important role during conidial dormancy.

Twelve stored mRNAs have been identified previously in the conidia of *A. nidulans *[13]. Among the corresponding genes, *CETA *is orthologous to a plant thaumatin-like PR5 gene, four genes encode metabolic enzymes (isocitrate lyase, transketolase, PEP carboxykinase, UDP-galactose epimerase), one is an ortholog of the glucose repressed gene *grg1 *and the six other genes are of unknown function. Recently Belaish and co-workers [43] analysed the two members of the thaumatin-like protein family in *A. nidulans, CETA *and *CALA*. If the single *cetaΔ *and *calaΔ *mutants were phenotypically identical to the wild type, the double *calaΔ/cetAΔ *mutant showed lethal conidial lysis suggesting that these two genes affect germination in a coordinated way.

In *N. crassa*, the set of genes that are the most expressed after 30 min are heat shock proteins responsible for the correct folding of the newly synthesized proteins [4]. In *A. fumigatus*, such expression of HSPs is not obvious. In spite of its thermophily, the number of annotated HSPs in the *A. fumigatus *genome is relatively low: 28 HSPs have been identified that belong mainly to the *HSP70 *(11 members) and the *HSP60 *(10 members) families; the other HSPs are 3 members of the HSP20/30 families and 3 singletons coding for an *HSP9*, *HSP12 *and *HSP90 *(Tekaia and Latgé, unpublished). Among these, 9 HSP genes are present on the membranes and only one of these (Afu2g16020) was over-expressed during germination of both one-week and one-year old conidia. Afu2g16020 codes for an ortholog of *TCP1 *that is an essential chaperone of *S. cerevisiae *involved in the maintenance of the actin cytoskeleton [44]. With the exception of the set of HSP genes, the expression of all the gene families of *N. crassa *which are up-regulated after 1 h incubation viz. amino acid biosynthesis; transcription, DNA processing, ribosome biogenesis, energy and transport were also up-regulated in *A. fumigatus*.

One interesting finding coming out from our study is the putative role of transposable elements in the activation of the conidial metabolism during exit from dormancy. In human, 45% of the genome is composed of retrotransposable elements [45]. Only a fraction is able to retrotranspose and the highest proportion is inactive because the coding sequence of most retrotransposons is truncated or heavily rearranged. In *A. fumigatus*, inactive LTR-retrotransposons also exist and have been used efficiently for strain typing [46,47]. Most of the transposable elements that have been annotated in *A. fumigatus *have however a complete coding sequence. Based on percentage of similarity, 56 DNA transposons (among which 19 Pogo/Taf1 and 26 Impala orthologs) and 38 retrotransposons (with 29 LINE-like elements) have been identified (Tekaia, unpublished). The data shown here suggested indeed that these transposable elements are transcripted in this fungal species. Transposition of elements has been described in many filamentous fungi. However, the only transposable element studied in *A. fumigatus *is *TAF1 *which is transcribed but did not transpose under different stresses (heat shock of mouse infection) [48]. Retrotransposon-derived sequences in humans are developmentally regulated and effective retrotransposition occurs in highly replicating cells (embryos, tumors) [49]. Data obtained here suggest that retrotransposition may be involved in regulating transcription of early developmental genes during germination and open new concepts in the role of transposable elements in molds.

## Conclusion

Transcriptomic data indicate that the exit from dormancy is associated with a shift from a fermentative metabolism to a respiratory metabolism as well as a trend toward immediate protein synthesis.

## Methods

### Strain, media and conidium production

*A. fumigatus *strain af293 was used throughout this work. For conidium production, this strain was inoculated to malt agar slants (2% malt; 2% agar; Becton, Dickinson and Company, Sparks, MD, USA) and incubated for 7 days at 37°C. As the slants were capped with a thin cigarette paper, the agar dried out and conidia were harvested by simply knocking on the glass tube on top of an aluminium foil. It was verified by light microscopy that the conidium pools were free of contaminating hyphae or conidiophores. The dry resting conidia were separated in two batches: the first batch was used immediately whereas the conidia of the second batch were stored dry in a beaker at RT for a year. Conidia were always recovered in a dry state because preliminary assays have shown that vortexing slants with a 0.05% detergent solution to recover conidia will solubilise nutrients from the agar medium of the slant that could impact on gene expression even in absence of morphogenetic changes during further incubation of the conidia at 37°C.

### Conidium germination

Conidia were suspended in 50 ml of an 0.05% Tween 20 aqueous solution, and added to a 1 l glass flask containing 500 ml of YPD (1% yeast extract, 2% peptone and 2% dextrose), and incubated at 37°C with agitation using a magnetic stirrer (750 rpm). The conidial density was 5 × 10^6 ^conidia ml^-1^. Samples were collected at 30 min intervals from 0 to 90 min. Controls with conidia incubated in water under the same conditions were also prepared.

### Extraction and purification of total RNA

For extraction of total RNA, conidia (at least 10^8^) were suspended in 500 μl of 0.1% SDS and added to screw cap microcentrifuge tubes containing 500 μl of glass beads (1 mm diameter glass beads; BioSpec Products Inc., Bartlesville, OK, USA) and 600 μl of phenol (pH 5; Prolabo, Fontenay-sous-bois, France). Tubes were vortexed vigorously using a Fastprep apparatus (3 × 30 sec; power 4.0; 4°C; MP Biomedicals, Illkirch, France). After centrifugation at 13,000 × *g *for 5 min at 4°C, the supernatant was extracted three times with 500 μl phenol (pH 5) and two times with 500 μl chloroform. Contaminant DNA was removed from samples by DNase treatment (GE Healthcare, United-Kingdom) according to the manufacturer's instruction. Total RNA was precipitated from supernatants by addition of 0.1 volume of a 3 M sodium acetate solution (pH 5.2) and 2.5 volumes of absolute ethanol, and incubated overnight at -20°C. Total RNA samples were pelleted by centrifugation, washed with 70% ethanol, dissolved in 50 μl of water and stored at -20°C. Total RNA concentration was calculated from the absorbance at 260 nm and the quality checked using BioAnalyzer Agilent technologies (Santa Clara, CA, USA).

### Genomic library construction, amplicons and macroarrays production

Genomic DNA was extracted from a 24 h *A. fumigatus *mycelial culture fractionated by nebulization. Fragments of 0.6 to 0.9 kb were collected by size-fractionation and cloned into the *Bst *XI restriction site of the high copy number vector pCDNA2.1 (Invitrogen, Carlsbad, CA, USA) using the *Bst *XI adapters method [50]. After transformation into *E. coli *and selection on LB-plates, about 10,243 individual clones were picked using a Qpix robot (Genetix Ltd, Queensway, New Milton, Hampshire, UK) and grown in 96-well plates in 200 μl volume. Plasmidic DNA was prepared in sufficient amounts for further use as templates for DNA sequencing, PCR reactions and conservation as DNA stock. A unique identification name was given to each clone [afr(number)(letter)(number)]. DNA sequences were compared against Nrprot (version 11.26), which is a NCBI non-redundant databank composed of Genbank CDS translations, PDB, Swissprot and PIR (585,527 sequences; 184,238,107 total letters) using blastx 2.1.2 (expect value for the first match ≤ 1E^-6^). About 7,000 selected sequences have been analyzed by different computer methods. i.e. a statistical analysis based on the hidden Markov model predicting STS which correspond to genes. Introns can be predicted and possible overlaps between STS have been also identified. These sequences, together with the annotations are accessible on a web-browsable database (go to "links" in the fungwall website) [51]. Amplification reactions of the inserts of each plasmid were performed in 96-well plates (Applied Biosystems, Foster City, CA, USA) in a 100 μl reaction volume containing 10 ng of plasmidic DNA, 1 U of Taq DNA polymerase (Roche, Basel, Switzerland), 0.4 μM each universal primer and 0.2 mM dNTPs. Reactions were cycled 25 times (94°C for 1 min; 55°C for 1 min; 72°C for 2 min) with one final cycle of 72°C for 7 min on a GeneAmp PCR system 9,700 (ABI, Foster City, CA, USA). Successful amplification of each PCR product was verified on agarose gels, and negative PCRs were repeated, resulting in the final amplification of PCR products distributed in forty five 96-well plates. DNA arrays used for hybridization experiments were constructed using a Qpix robot (Genetix Ltd, Queensway, New Milton, Hampshire, UK) and Performa^II ^nylon membranes (Genetix Ltd, Queensway, New Milton, Hampshire, UK) as described in Milohanic and co-workers [52].

Among the 5,664 PCR products spotted onto membranes, 4,242 corresponded to 3,182 genes annotated in the Central Aspergillus Data Repository website [53], representing approximately one third of the whole *A. fumigatus *genome (see Additional file 13), whereas 1,422 corresponded to unknown or unmatched sequences.

### cDNA labelling and hybridization to DNA arrays

cDNA probes were synthetized and labelled using 30 μg of total RNA mixed with 2 μl of oligo (dT)_12–18 _(Invitrogen, Carlsbad, CA, USA) and heated at 70°C for 10 min. After chilling on ice, 1.5 μl of dNTP mixture containing dATP (10 mM), dGTP (10 mM), dTTP (10 mM) and dCTP (625 μM; all from GE Healthcare, UK), 1 μl DTT 0.1 M (Invitrogen, Carlsbad, CA, USA), 5 μl of [α-^33^P]dCTP (10 mCi ml^-1 ^with a specific activity of 3000 Ci mmol^-1^; Perkin-Elmer, Waltham, MA, USA) and 1 unit reverse transcriptase (Superscript™ II RNase Transcriptase; Invitrogen, Carlsbad, CA, USA) were added to the solution which was then incubated overnight at 42°C.

The membranes were rehydrated in 2 × SSC and pre-hybridized at 68°C using roller bottles with 10 ml of hybridization solution (5 × SSC; 5 × Denhardt solution; 1% SDS; 10 μg/ml sperm salmon DNA). The labelled-cDNA probes were boiled for 10 min and added to 5 ml of new hybridization solution. Hybridization was carried out for 16–18 h at 68°C. Then the membranes were washed three times for 20 min at RT with 1 l of 0.2 × SSPE supplemented with 0.1% SDS, and once for 5 min at RT with 2 × SSC. The membranes were finally wrapped in plastic bags and exposed to a Phosphoimager screen (GE Healthcare, UK) for 24 h. Undried membranes were stripped with 1 l of boiling dehybridization solution (1% SDS) 4 × 15 min.

### Experimental design, image acquisition and data analysis

In our experimental design, macroarray hybridization experiments using cDNAs from one-week old conidia were performed with conidia incubated in water or YPD for 0, 30, 60 and 90 min. When using the one-year old conidia, macroarray hybridization experiments were performed with RNA isolated from conidia only incubated 0 and 30 min in YPD medium. Four cDNA probes for each time point were derived from independent conidium batches and the total experiment was repeated twice (8 replicates per condition). Probes were hybridized on different filters. All filters were used up to four times.

Digital hybridization images from exposed phosphoimager screens were captured by using the STORM 860 phosphoimager (GE Healthcare, UK), and to identify transcripts analyzed by the ArrayVision™ 6.0 software (GE Healthcare, UK). All data were analyzed after normalization of each spot. The normalized value of a spot was calculated from the spot signal minus the background level of hybridization (determined from the intensity of the signal surrounding each spot), divided by the median value of all the signals detected on the filter. Eight replicates of the two biological repetitions were used. To identify genes with stored transcript in the resting conidium, a threshold value was calculated based on the empty spots of the membrane (385 spots received only buffer without PCR product). This threshold corresponded to the mean of the intensity values recorded for all empty spots + (2 × empty spots standard deviation). One particular spot and its associated gene were declared positive for a mean value greater than the threshold value using the one-tailed Z-test at 0.023 level. To determine induction or repression of gene expression, all spots intensities were compared among the filters corresponding to the following paired time point (0 and 30 min; 30 and 60 min; 60 and 90 min). Genes whose expression level was altered more than twice were considered differentially expressed when they both fit the ratio and the Student's two-sample test (*p *value cut-off of 0.05) as described in the software Bioplot available from the biochips platform of the Toulouse Midi-Pyrenees Genopole [54].

Gene assignment to Gene Ontology (GO) category was provided by Jane Gilsenan from University of Manchester School of Medicine, U.K. and freely accessible at the ftp server of the university. The GO hierarchy was obtained from the Gene Ontology AmiGO website [55].

### Northern blot and RT-PCR analysis

These two methods were used to confirm the expression data of the macroarrays. For Northern blots, 20 μg of total RNA was separated by electrophoresis on a denaturing 1.0% agarose gel containing formaldehyde and blotted to a Hybond-N+ nylon membrane (GE Healthcare, UK). An approximately 500 bp PCR-amplified insert of genomic DNA was labelled using the random Readyprime II labelling kit following the manufacturer's instructions (GE Healthcare, UK). Amplification reactions of the inserts of each gene were performed in a 50 μl reaction volume containing 1 μg of genomic DNA, 1X PCR buffer, 1 unit of DNA polymerase (Advantage II, Clontech Laboratories Inc, Mountain View, CA, USA), 0.4 μM of each primer (primers are listed in Additional file 14) and 0.2 mM dNTPs. After an initial cycle at 94°C for 1 min, reactions were cycled 30 times (94°C for 30 s; 68°C for 3 min) in a thermocycler (BioRad, Hercules, USA). RNA-gel blots were hybridized overnight, and the membranes were washed three times for 20 min at RT with 1 l of 0.2 × SSPE supplemented with 0.1% SDS, and once for 5 min at RT with 2 × SSC. Signals were detected with a phosphoimager screen. For RT-PCR assays, RNA samples were purified with the RNeasy mini kit (Qiagen, Hilden, Germany), according to the manufacturer's instructions. It was verified that RNA obtained with this procedure was free of genomic DNA using primers 5afu4g11580 and 3afu4g11580 that encompasses three introns of the SOD2 gene. The presence of a DNA contamination will be shown by differences in amplicon sizes of the SOD2 gene with a size of 907 bp obtained using genomic and 611 bp using cDNA templates. Total RNA (2 μg) was reverse transcribed in the presence of 1X first-strand buffer, Oligo(dT)_12–18 _(500 ng), 0.5 mM dNTPs, 0.01 M DTT, 2 U RNaseOUT and 10 U SuperScript II RT in a final reaction volume of 20 μl. Reactions were carried out at 42°C for 50 min, followed by a 15 min at 75°C. Different dilutions of cDNA (equivalent to 0.00004 to 10% v/v of starting material depending on the gene expression level) were amplified with 1X Advantage 2 Polymerase Mix (Clontech Laboratories Inc, Mountain View, CA, USA) according to the manufacturer's instructions, and in the presence of 0.4 μM of each couple of primers. The primer sequences were determined using the software Primer 3 available on-line [56]. Primers were always selected according to the following parameters: 30 bases length, T_m _at 70°C, length of amplification product between 500 and 600 bp. A first cycle of 1 min at 95°C, was followed by 30 sec at 95°C and 3 min at 68°C for 30 cycles.

### Purification of plasma membranes and western blot analysis

Plasma membranes were prepared from resting and germinating conidia basically as described by Burghoorn and co-workers [16]. Conidia were collected by centrifugation and suspended in homogenization buffer (0.4 M sucrose/2 mM EDTA/25 mM imidazole-HCl, pH 7.0). Conidia were broken with glass beads using a FastPrep apparatus (see above for breakage conditions). After cell disruption, the cell homogenate was centrifuged at 80,000 × *g *for 18 h in a 1.1 M – 2.25 M sucrose gradient in a 2 mM EDTA/25 mM imidazole-HCl (pH 7) solution and the membrane pellet proteins were separated by 10% SDS-PAGE and blotted onto nylon membrane in a blotting apparatus according to the manufacturer's instructions. After blocking, anti-PMA1 antibody (kind gift from D.S. Perlin, PHRI, Newark, USA) was diluted 1/5,000 and labelled with an anti-rabbit peroxidase conjugate diluted 1/1,000. Peroxidase labelling was performed using ECL chemiluminescence kit (GE Healthcare, UK) as described by the manufacturer.

## Authors' contributions

CL^1 ^and JPL conceived the project, and directed its design and execution. CL^1 ^was responsible for all of the experiments. CH and CL^3,5 ^performed PCR amplifications and robotics set-ups. PG was responsible for preparing and sequencing the clones of the *A. fumigatus *DNA library. JYC was in charge of the microarray platform for transcriptome data acquisition. JPD participated to data acquisition. SS and JMF were responsible for all statistical analysis. JPL and CL^1 ^drafted the manuscript. All authors read and approved the final manuscript.

## Supplementary Material

Additional file 1 **Genes constitutively transcribed in the dormant conidia.** Click here for file

Additional file 2 **Genes up-regulated and down regulated during the first 30 min of incubation at 37°C in a YPD medium of one-week old conidia.** The genes without transcript at time 0 and that are up-regulated during this incubation time are also shown. Genes marked with an asterisk are undetectable after 30 min of germination. Click here for file

Additional file 3 **Repartition of up- and down-regulated genes among GO categories for one-week and one-year old conidia during the first 30 min of germination.** Click here for file

Additional file 4 **List of modulated genes repartition among GO categories for one-week old conidia during the first 30 min of germination.** Click here for file

Additional file 5 **Genes up-regulated and down regulated during the first 30 min of incubation at 37°C in a YPD medium of one-year old conidia.** The genes without transcript at time 0 and that are up-regulated during this incubation time are also shown. Click here for file

Additional file 6 **List of modulated genes repartition among GO categories for one-year old conidia during the first 30 min of germination**. Click here for file

Additional file 7 **List of transcription factors up-regulated during the first 30 min of germination.** Click here for file

Additional file 8 **Genes which expression change between 30 and 60 and between 60 and 90 min of incubation of one week old conidia in YPD medium**. Genes marked with an asterisk are undetectable after 60 and 90 min of germination. Click here for file

Additional file 9 **Genes belonging to the four clusters A-D which expression changed between 0 to 90 min of incubation of one week old conidia in YPD medium.** Click here for file

Additional file 10 **Northern blots of genes from Clusters A **(A)**, B **(B) **and C **(C)**.(D) **total RNA stained with ethidium bromide are shown as loading control. Click here for file

Additional file 11 **RT-PCR assays showing the changes of expression of 76 genes.** Selection of these genes was based on their expression pattern seen in the macroarray experiments. **A: **Controls with *SOD2 *primers showing the absence of DNA contamination in the cDNA samples used for the RT-PCR assays. G Genomic DNA; cDNA from samples obtained after 0 (0) and 30 min (30) incubation in a rich medium. **B: **Three genes randomly selected among genes which expression did not vary during the first 30 min. germination. **C1: **Five genes the most up-regulated in both one-week old and one-year-old conidia. **C2: **Five genes the most up-regulated in one-week old conidia with an expression not modified in one-year old conidia. **C3: **Five genes with an expression not modified in one-week old conidia and an expression the most up-regulated in one-year old conidia. **D1: **Five genes the most down-regulated in both one-week old and one-year old conidia. **D2: **Five genes the most down-regulated in one-week old conidia and showing no variation of expression in one-year old conidia. **D3: **Five genes with an expression not modified in one-week old conidia and an expression the most down-regulated in one-year old conidia. **E: **Up-regulation of four genes involved in amino acid biosynthesis in one-week old conidia. **F: **Down-regulation of genes involved in fermentation metabolism in one-week old conidia. **G: **Down-regulation of glycosylhydrolases putatively involved in cell wall softening in one-week old conidia. Click here for file

Additional file 12 **Western blot showing the presence of the Pma1p in the swollen conidia.** Lane A: Coomassie blue staining of the proteins extracted from the resting (A1) and germinated conidia (A2). B1 and B2: Western blot of the protein extracts of the A1 and A2 with an anti-pma1 antibody. Click here for file

Additional file 13 **List of genes spotted in the array.** Click here for file

Additional file 14 **List of primers used for RT-PCR amplification.** Click here for file
