# Supplementary figures and images for: Transcriptomic analysis of the exit from dormancy of Aspergillus fumigatus conidia
Source: BMC Genomics. 2008 Sep 16;9:417. doi: 10.1186/1471-2164-9-417 (PMC2556354; doi:10.1186/1471-2164-9-417)

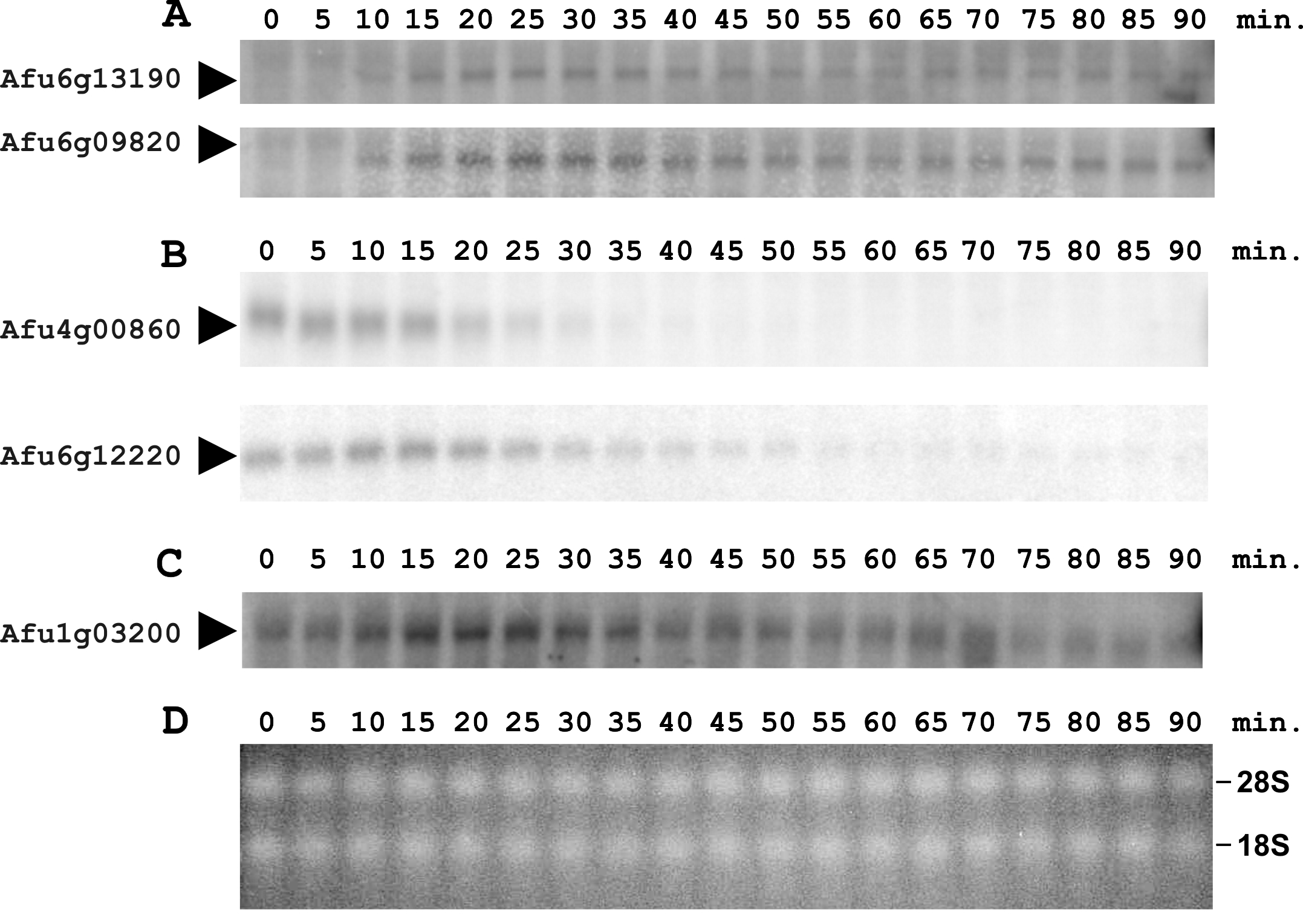

Supplement: Additional file 10 — Northern blots of genes from Clusters A (A), B (B) and C (C).(D) total RNA stained with ethidium bromide are shown as loading control. [file 1471-2164-9-417-S10.jpeg]

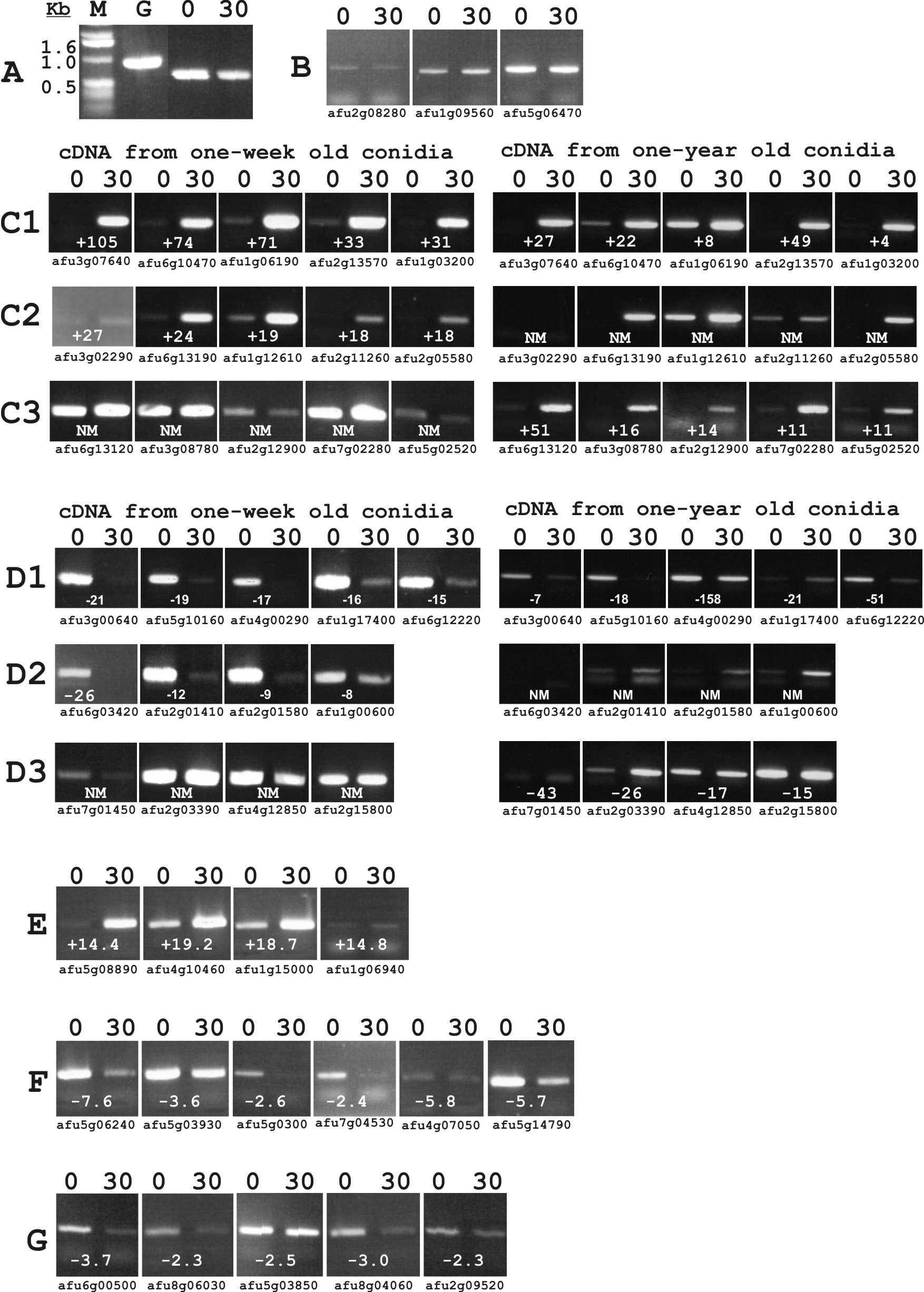

Supplement: Additional file 11 — RT-PCR assays showing the changes of expression of 76 genes. Selection of these genes was based on their expression pattern seen in the macroarray experiments. A: Controls with SOD2 primers showing the absence of DNA contamination in the cDNA samples used for the RT-PCR assays. G Genomic DNA; cDNA from samples obtained after 0 (0) and 30 min (30) incubation in a rich medium. B: Three genes randomly selected among genes which expression did not vary during the first 30 min. germination. C1: Five genes the most up-regulated in both one-week old and one-year-old conidia. C2: Five genes the most up-regulated in one-week old conidia with an expression not modified in one-year old conidia. C3: Five genes with an expression not modified in one-week old conidia and an expression the most up-regulated in one-year old conidia. D1: Five genes the most down-regulated in both one-week old and one-year old conidia. D2: Five genes the most down-regulated in one-week old conidia and showing no variation of expression in one-year old conidia. D3: Five genes with an expression not modified in one-week old conidia and an expression the most down-regulated in one-year old conidia. E: Up-regulation of four genes involved in amino acid biosynthesis in one-week old conidia. F: Down-regulation of genes involved in fermentation metabolism in one-week old conidia. G: Down-regulation of glycosylhydrolases putatively involved in cell wall softening in one-week old conidia. [file 1471-2164-9-417-S11.jpeg]

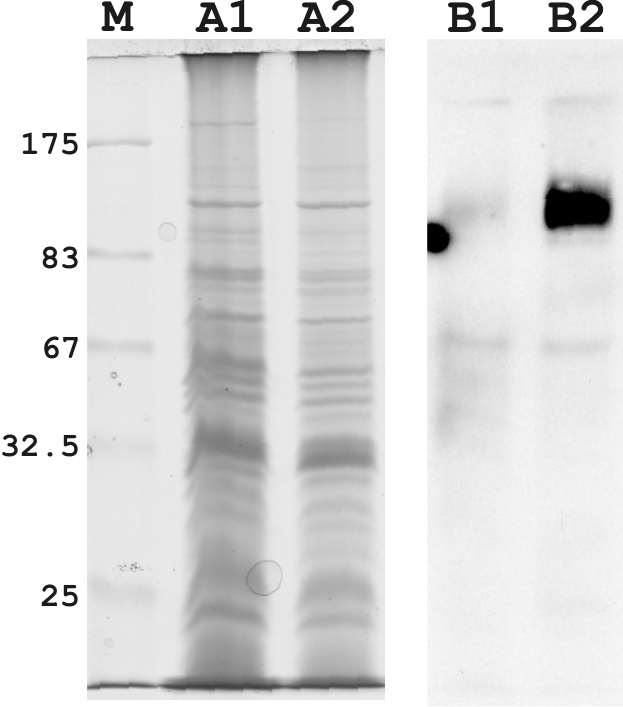

Supplement: Additional file 12 — Western blot showing the presence of the Pma1p in the swollen conidia. Lane A: Coomassie blue staining of the proteins extracted from the resting (A1) and germinated conidia (A2). B1 and B2: Western blot of the protein extracts of the A1 and A2 with an anti-pma1 antibody. [file 1471-2164-9-417-S12.jpeg]
